# Supplementary material for: Quantification of differential gene expression by multiplexed targeted resequencing of cDNA
Source: Nat Commun. 2017 May 5;8:15190. doi: 10.1038/ncomms15190 (PMC5424154; doi:10.1038/ncomms15190)
Supplement: Supplementary Information — Supplementary figures, supplementary tables, supplementary methods and supplementary references. [file ncomms15190-s1.pdf]

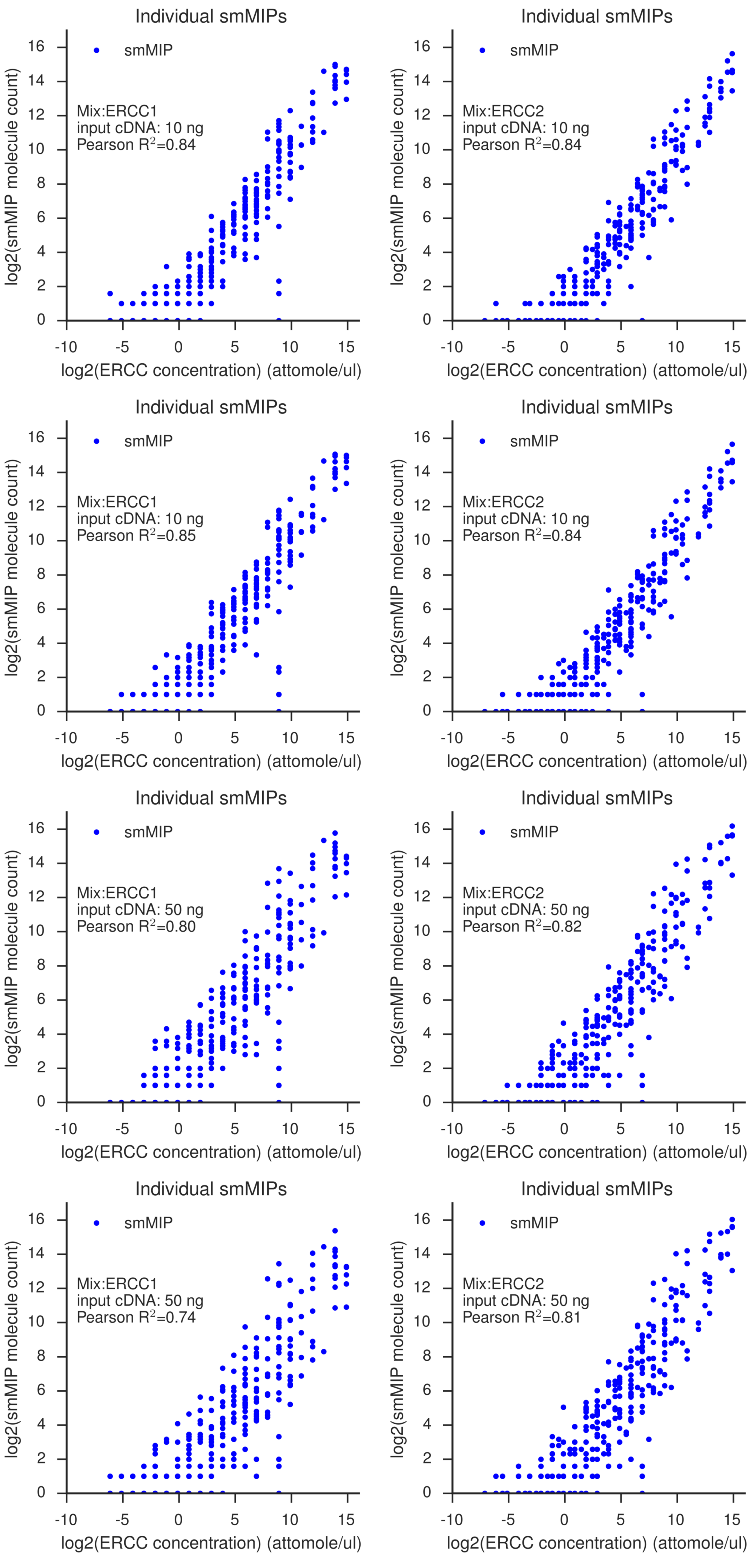

Supplementary Figure 1 Normalized smMIP molecule counts of individual smMIP probes vs known ERCC transcript concentrations. Each plot corresponds to a technical replicate.

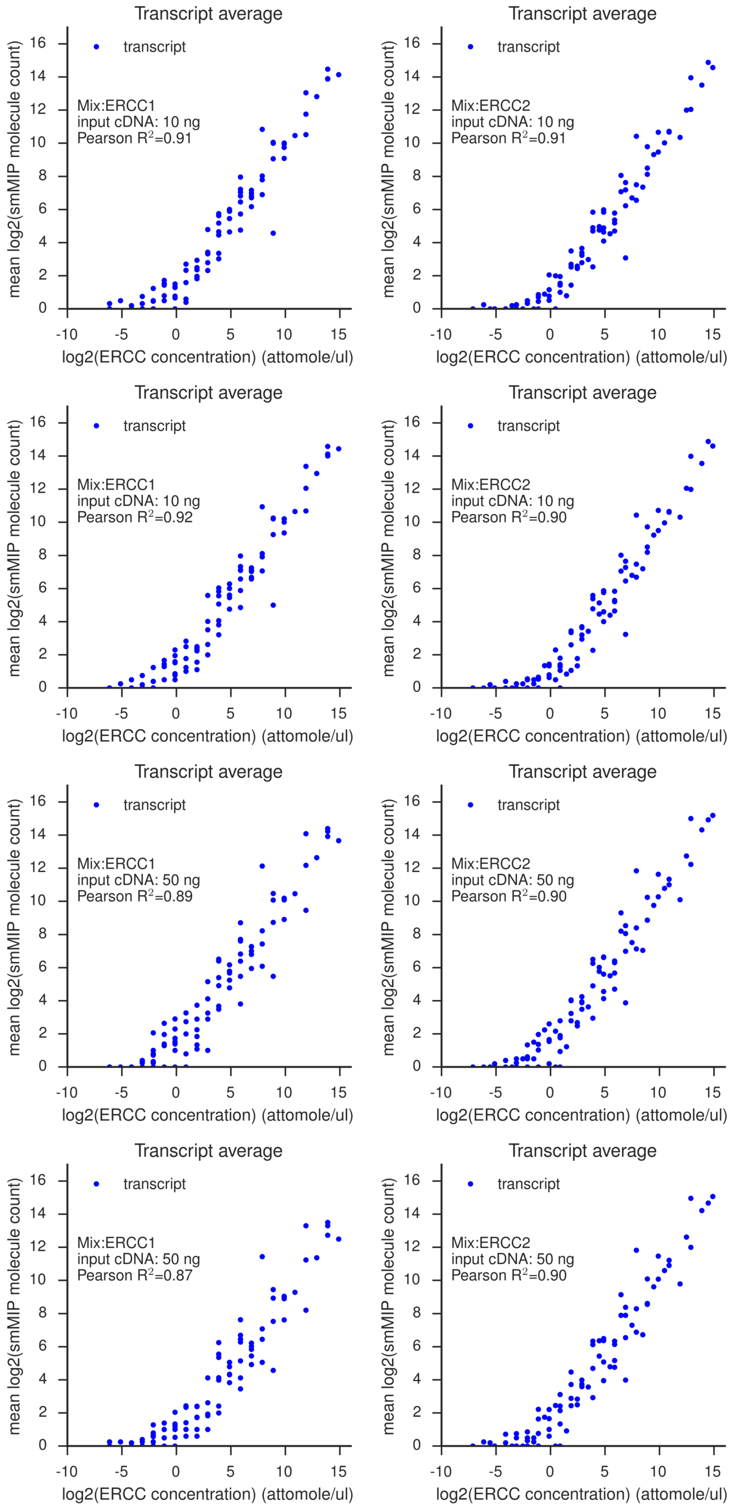

Supplementary Figure 2 Normalized smMIP molecule counts averaged per ERCC transcript vs known ERCC transcript concentrations. Each plot corresponds to a technical replicate.

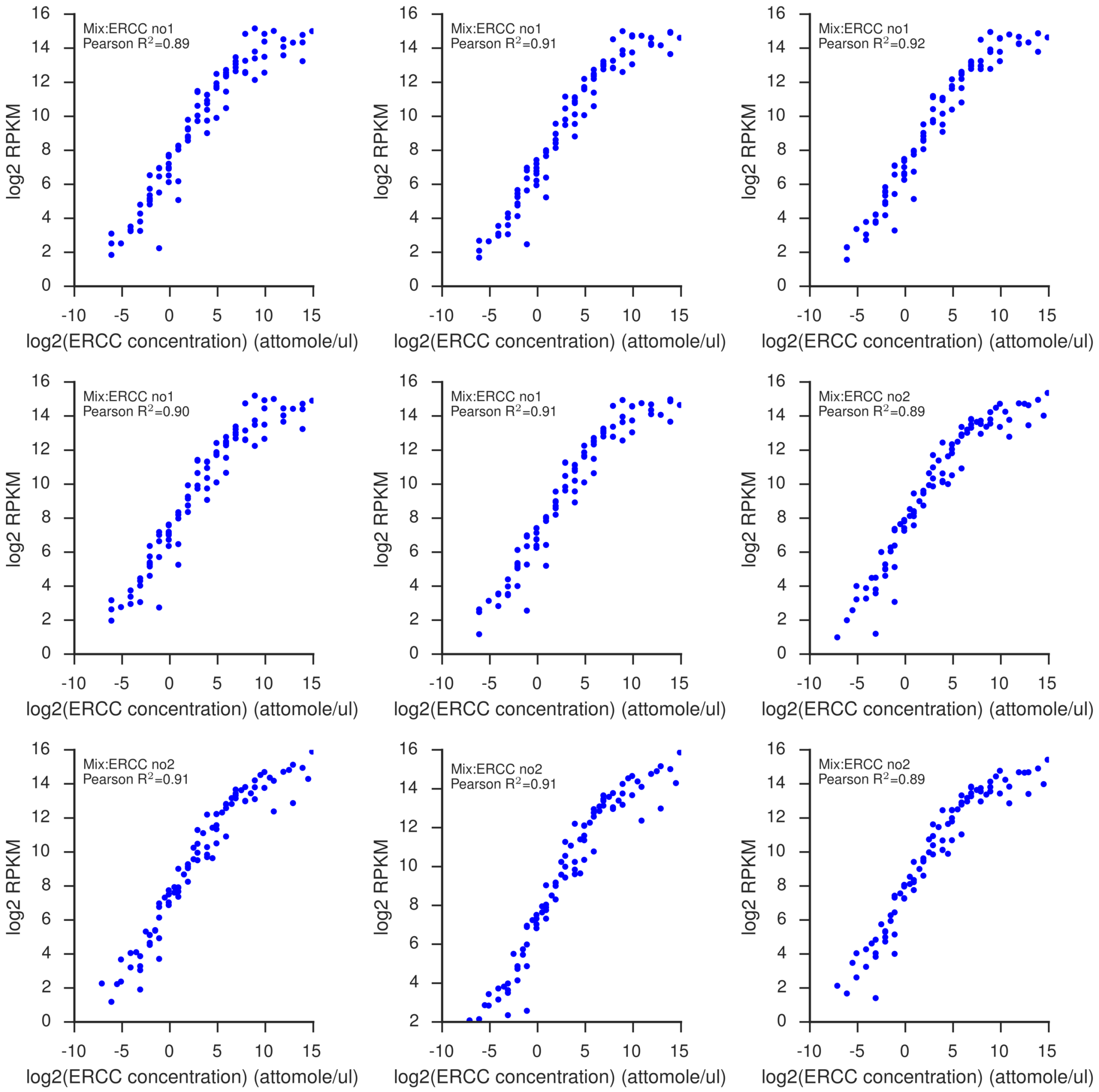

Supplementary Figure 3 Normalized transcript read counts (RPKM) of the CaptureSeq targeted RNA-Seq method[1] vs known ERCC transcript concentrations. Data was previously published[1] and downloaded from GEO accession GSE61474**.** Each plot corresponds to a technical replicate.

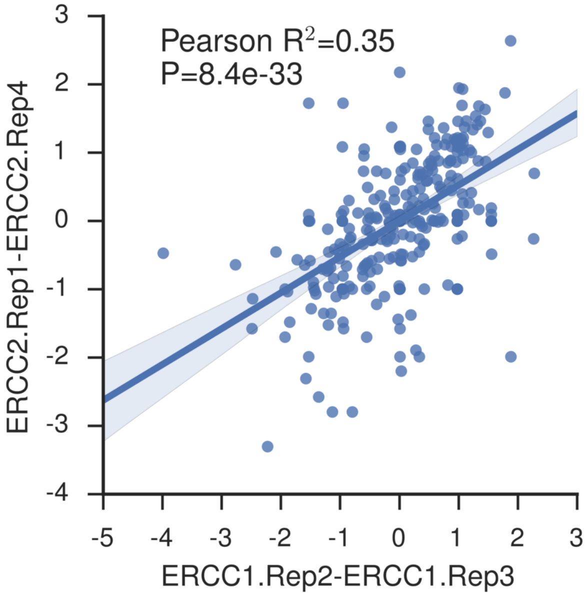

Supplementary Figure 4 Differences in log-normalized read counts between replicates of the same condition are correlated across conditions, indicating the present of systematic smMIP bias not associated with biological differences.

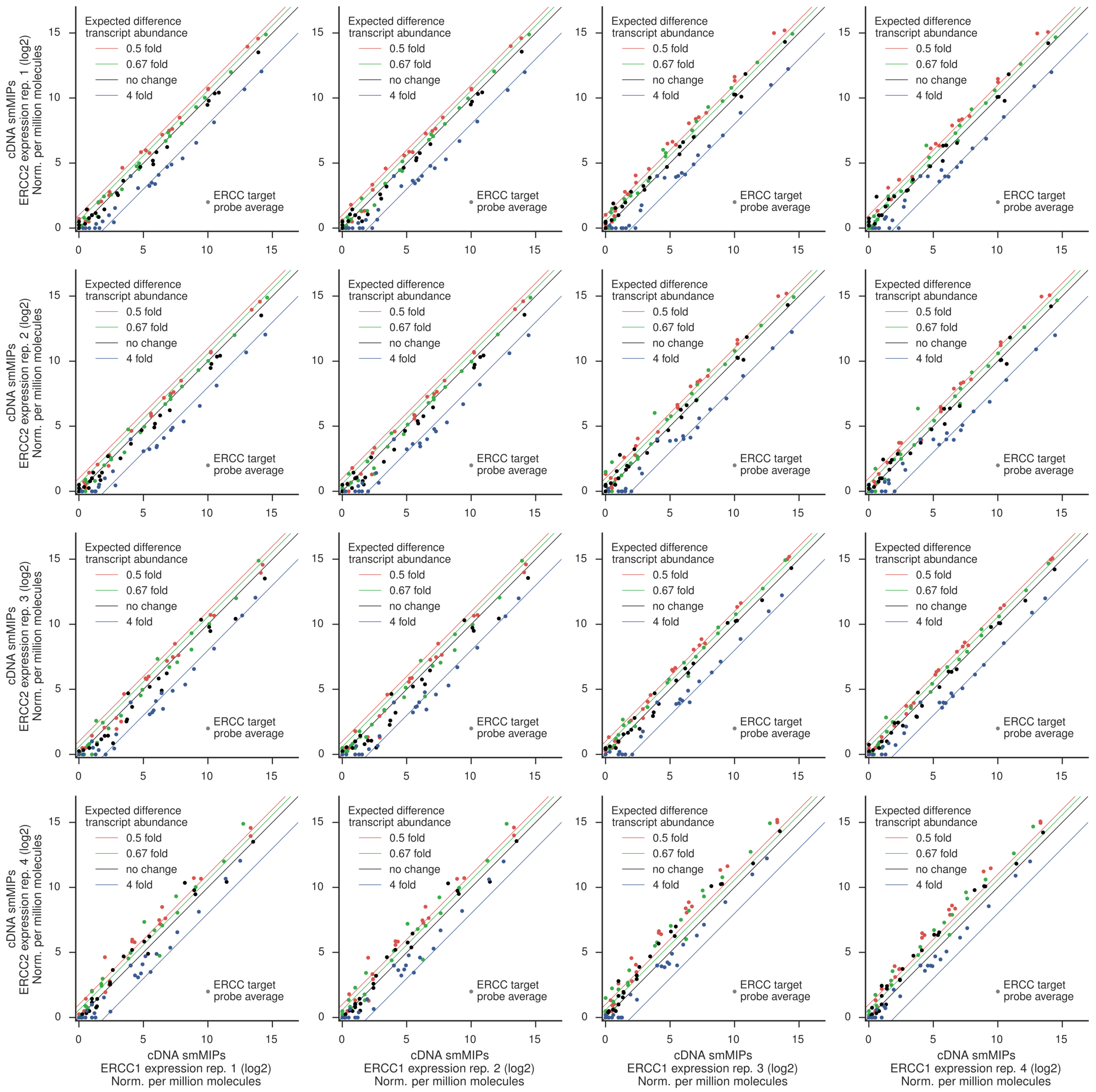

Supplementary Figure 5 Comparison of cDNA-smMIP normalized molecule counts for individual capture replicates from ERCC1 cDNA with individual capture replicates for ERCC2 cDNA. Each dot represents a transcript, where the cDNA-smMIP expression value was calculated as the average normalized molecule count of smMIPs targeting a given transcript, and is colored according to the expected fold-change (which is based on the known concentrations of the transcripts in respectively the ERCC1 RNA mix and the ERCC2 RNA mix). Note that these counts have not been estimated or corrected using our Bayesian model.

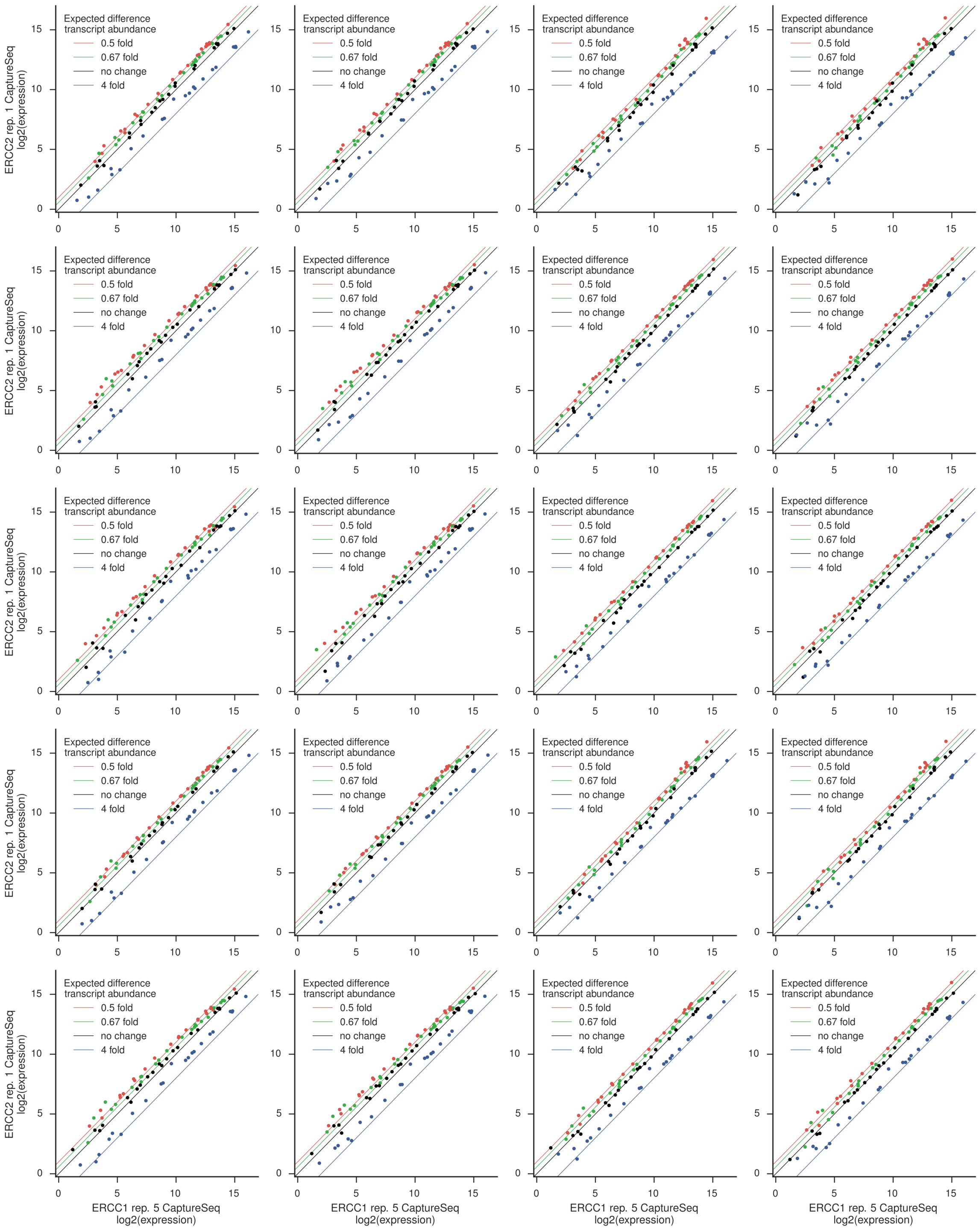

Supplementary Figure 6 Comparison of CaptureSeq normalized read counts (RPKM) from samples from ERCC1 RNA with samples from ERCC2 RNA. Each dot represents an ERCC transcript, and is colored according to the expected fold-change.

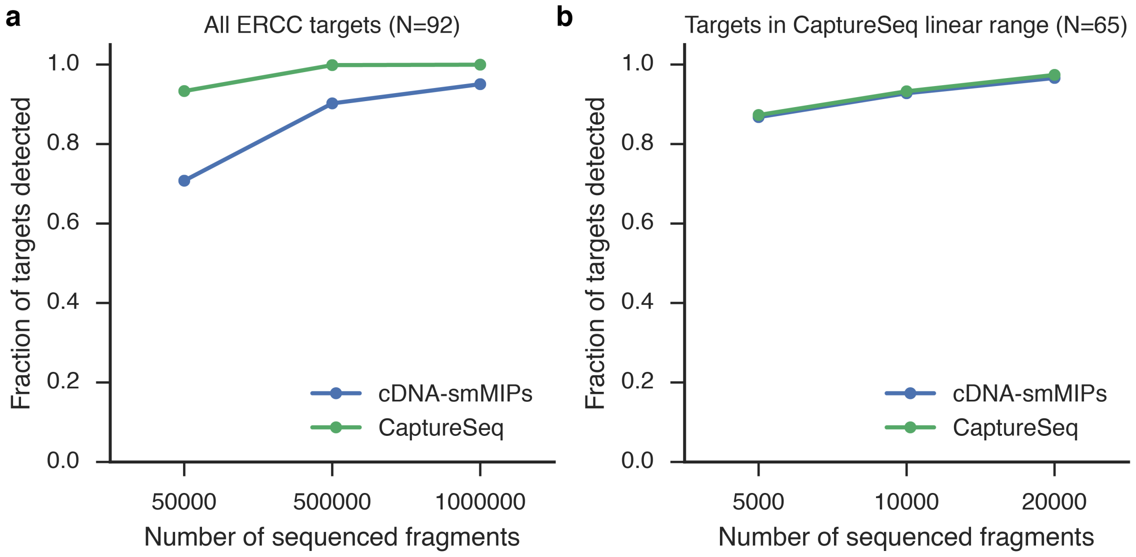

Supplementary Figure 7 Comparison of transcript detection sensitivity of cDNA-smMIPs and CaptureSeq. **a**) Comparison using all ERCC standards. Detection required 1 read mapping to the transcript. **b**) Comparison on the subset of transcripts where CaptureSeq quantification is linear (range < 100 attomol/ul). See Supplementary Fig. 3 and also Supplementary Fig. 5 in reference[1].

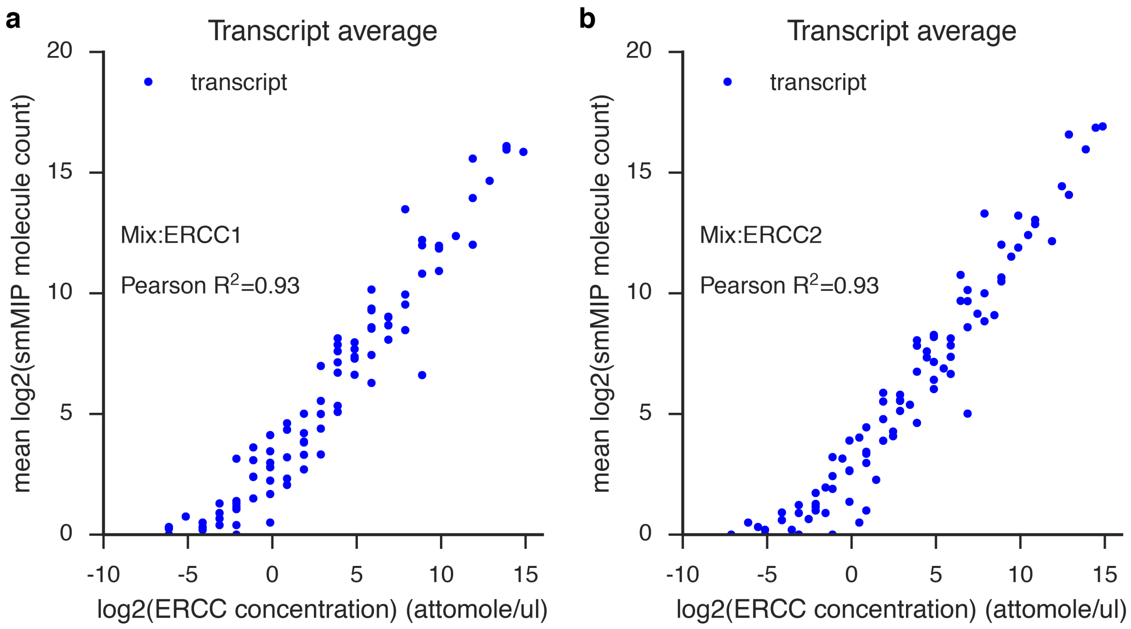

Supplementary Figure 8 Average transcript cDNA-smMIPs molecule counts obtained by pooling the counts from four technical cDNA-smMIP capture replicates for a) ERCC1 transcripts and b) ERCC2 transcripts. The increased read count shows that the deflection point at low concentrations observed in Supplementary Fig. 2 is diminished.

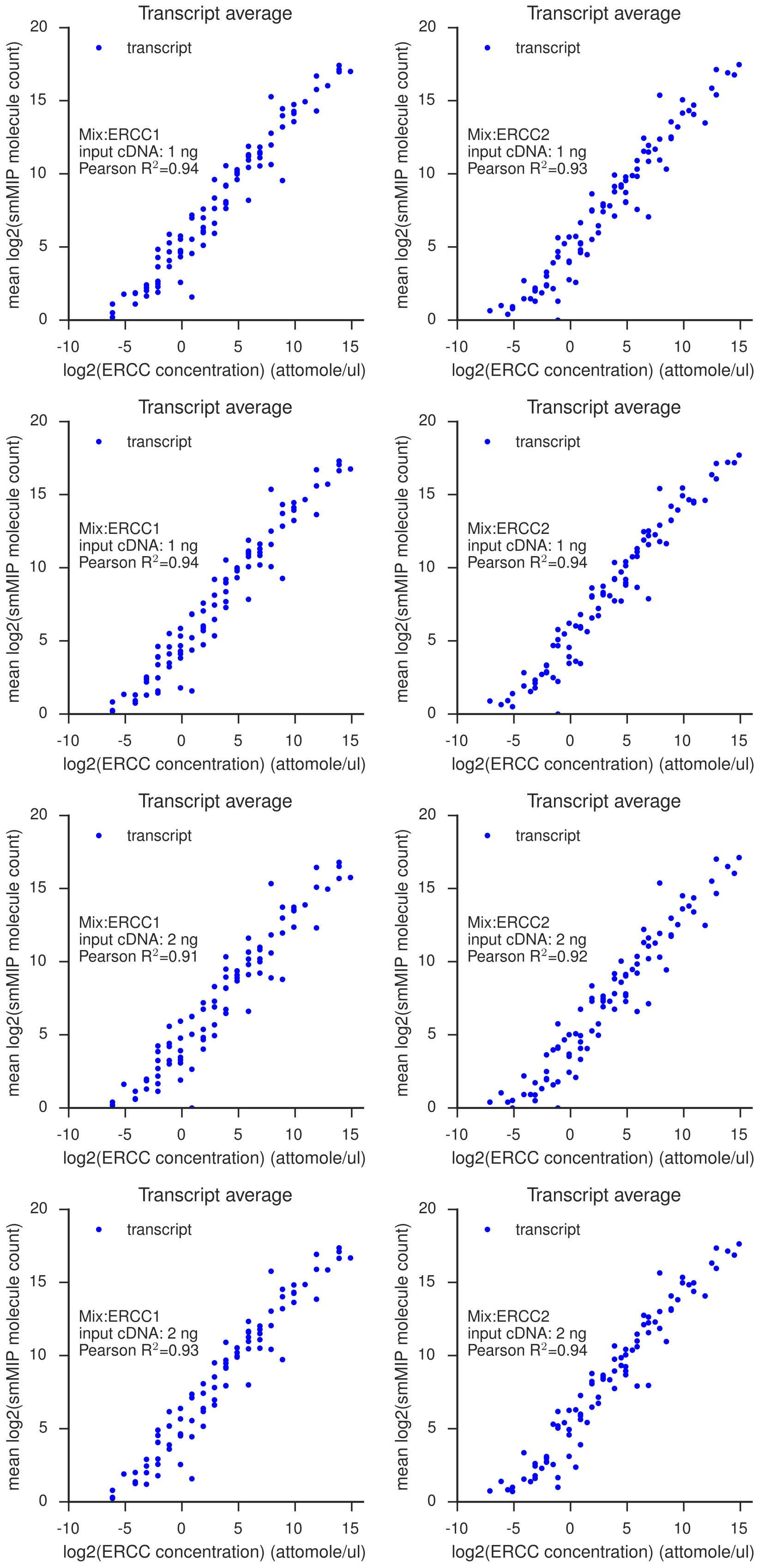

Supplementary Figure 9 cDNA-smMIPs average transcript molecule count vs known concentration at increased sequencing depth. To investigate quantification accuracy at low concentrations, cDNA from ERCC transcripts only (i.e. without PBMC RNA) was targeted with cDNA-smMIPs and sequenced at high total coverage. Each plot corresponds to a technical replicate.

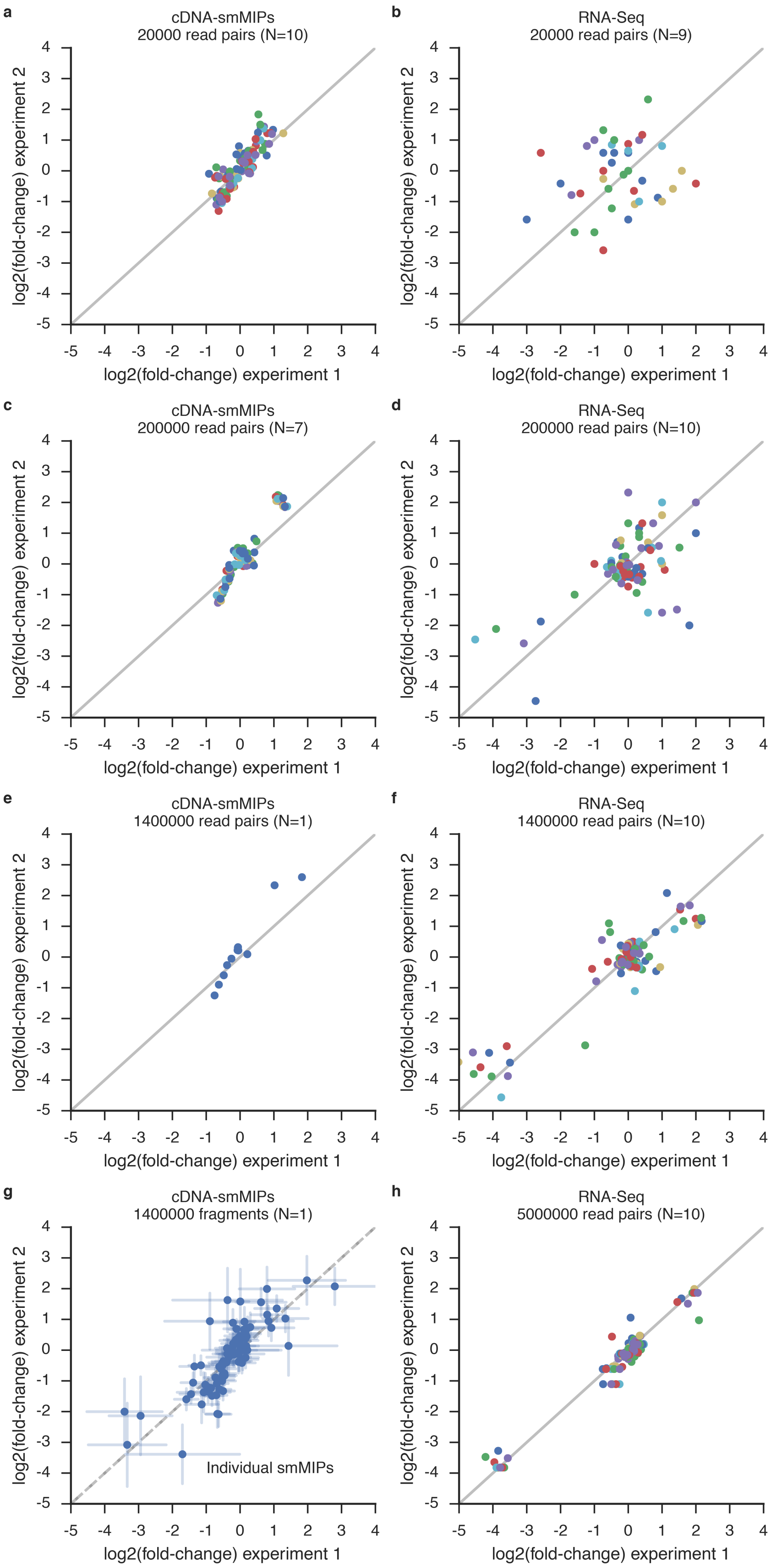

Supplementary Figure 10 Comparison of reproducibility of differential expression estimates of cDNA-smMIPs and RNA-Seq at the same depth of sequencing. Non-overlapping subsets of respectively 20,000 read pairs (panel **a**, **b**) 200,000 read pairs (panel **c**, **d**) 1,400,000 read pairs (panel **e**, **f**) and 5,000,000 read pairs (RNA-Seq only, panel **h**) were subsampled for two biological samples in two separate experiments. Panel **g** shows fold-change estimates for individual cDNA-smMIPs with corresponding 95% confidence intervals estimated by our statistical model.

Note 1. The individuals used for the RNA-Seq data are different from the individuals used for the cDNA-smMIPs data; consequently the fold-changes can only compared within the same technology.

Note 2. As the cDNA-smMIP libraries were not sequenced as deeply as the whole-transcriptome RNA-Seq libraries, only 1 replicate could be generated in panel **e** for cDNA-smMIPs.

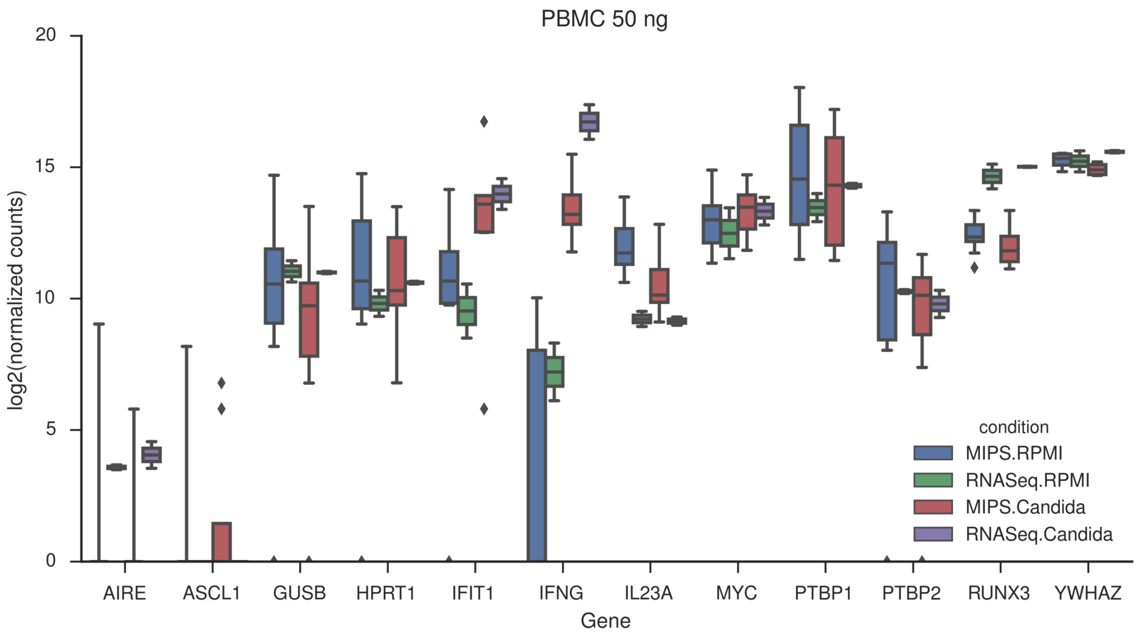

Supplementary Figure 11 Comparison of cDNA-smMIP normalized molecule counts with normalized read counts (RPKM) for RNA-Seq data for PBMC stimulation experiment (Fig. 4 in main text). For smMIPs, boxplots are over smMIPs and replicates (two per condition); for RNA-Seq data, boxplots are over gene RPKMs of different replicates. Only data from cDNA-smMIP captures with 50 ng input were included in the analysis (see **Supplementary Table 5**).

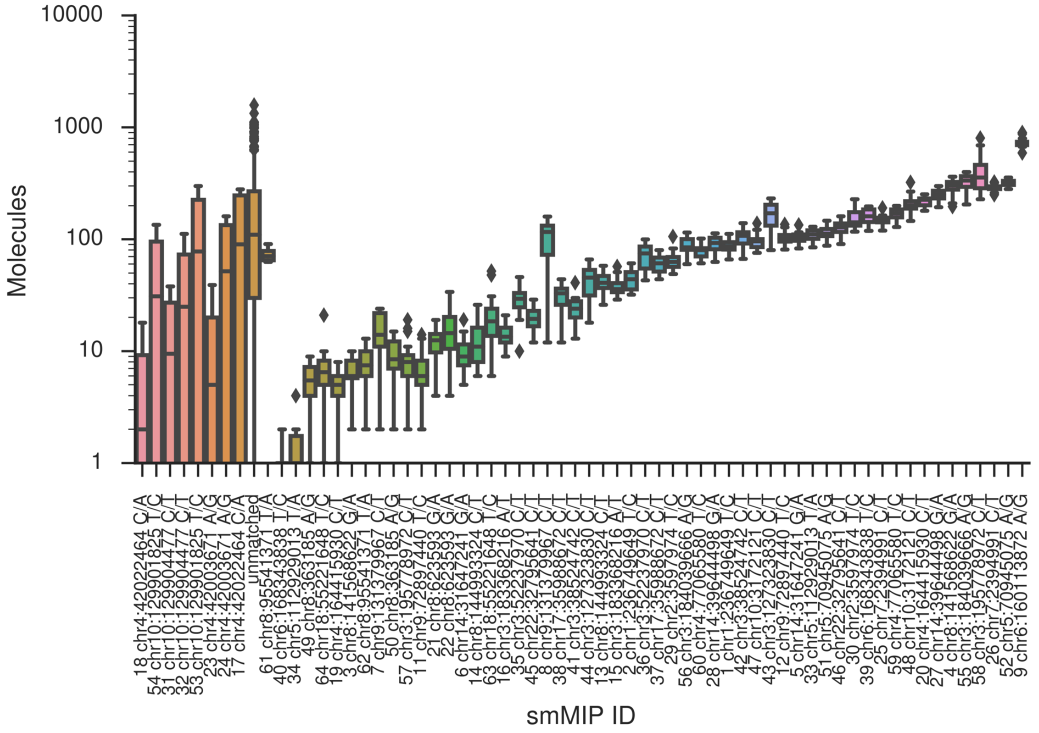

Supplementary Figure 12 Number of molecules observed for each smMIP in K562/HEK293 allelic ratio experiment. ‘unmatched’ represents UMIs from reads that could not be matched to a known smMIP based on the ligation or extension probe sequence. Statistics are also provided in

Supplementary Table 6.

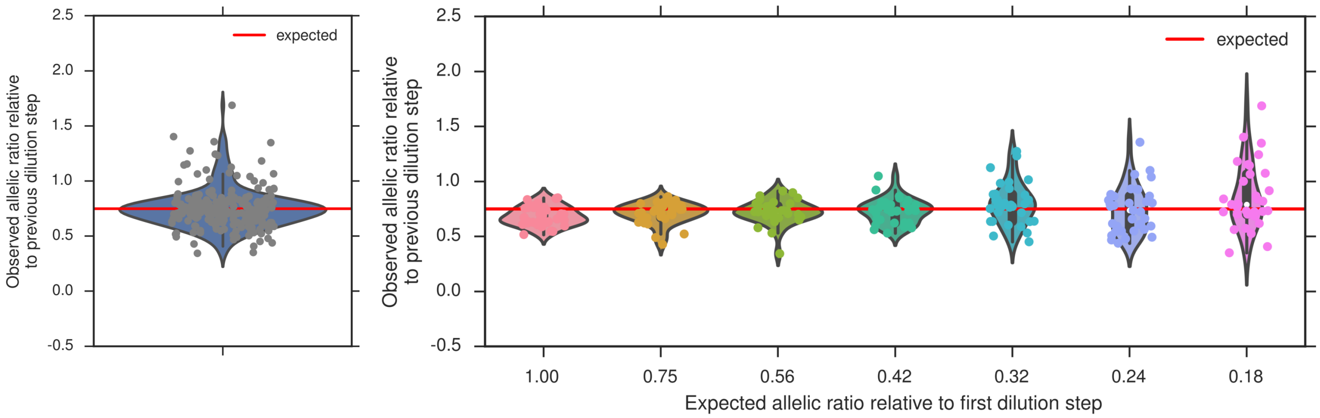

Supplementary Figure 13 Accuracy of estimation of allelic ratios with cDNA-smMIPs in a K562/HEK293 serial dilution experiment. In each dilution step, 75 ul of the dilution of the previous step is combined with 25 ul of HEK293 cDNA, resulting in an exponential decrease of the K562 allele 0.75^d. 64 smMIPs were designed targeting 32 SNPs for which the K562 and HEK293 were homozygous for the opposite allele.

A) The relative allelic ratio of subsequent dilution steps, across all dilution steps. For a given smMIP and dilution step *d*, the allelic ratio is defined as the fraction AR = $N_{K562}/(N_{K562+}N_{HEK293})$. The relative allelic ratio of dilution step *d* is then given by AR(d) / AR(d-1). This value is always expected to be 0.75, regardless of the initial expression ratios of the target transcript in K562 and HEK293 cells.

B) The relative allelic ratio stratified by dilution step. The horizontal axis represents the expected allelic ratio relative to the first dilution step (i.e., AR(d)/AR(1)), and illustrates that the absolute difference in the allelic ratio of subsequent dilution steps (AR(d)/AR(d-1), vertical axis) also becomes exponentially smaller. Given that the ratio is estimated from molecule counts, the variation in the allelic ratio relative to the *previous* dilution step becomes increasingly larger as the number of dilutions increases. The red line indicated the expected allelic ratio relative to the previous dilution step.

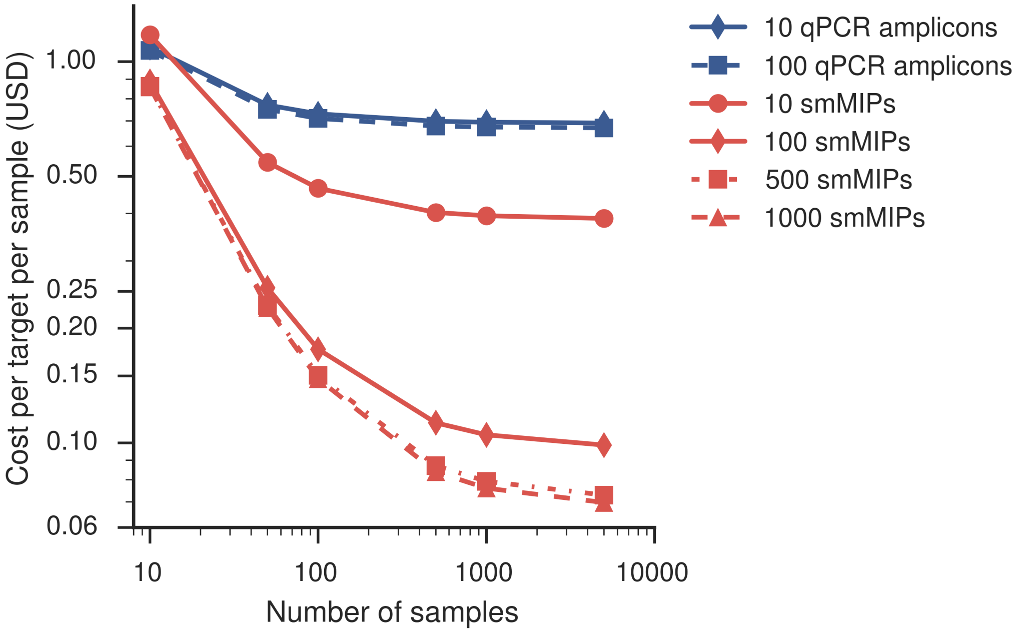

Supplementary Figure 14 Cost comparison of cDNA-smMIPs with qPCR

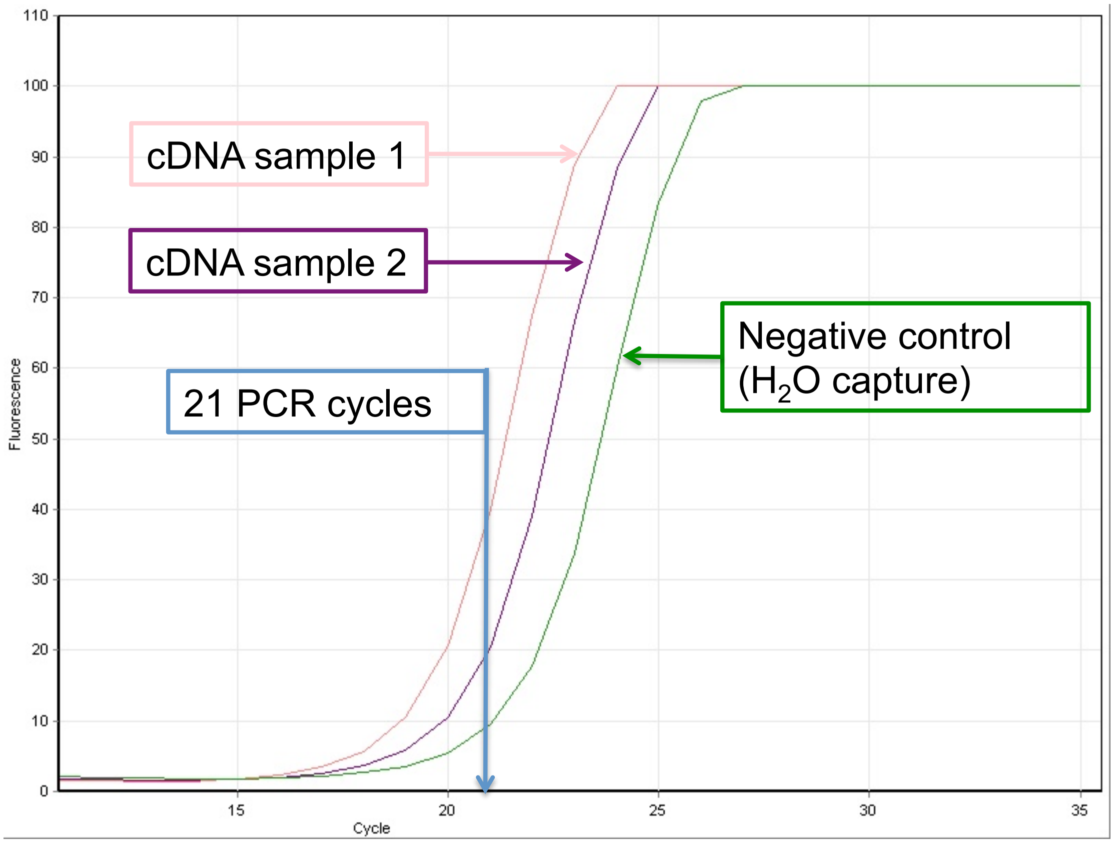

Supplementary Figure 15 RT-PCR results are used to determine the optimal amount of PCR cycles. Choose the amount of cycles when captured cDNA samples provide a fluorescent signal, but the captured H_2O control is negative. In this example 21 PCR cycles are sufficient. Negative control also produce a PCR product, as circularized but empty smMIPs can be amplified during PCR.

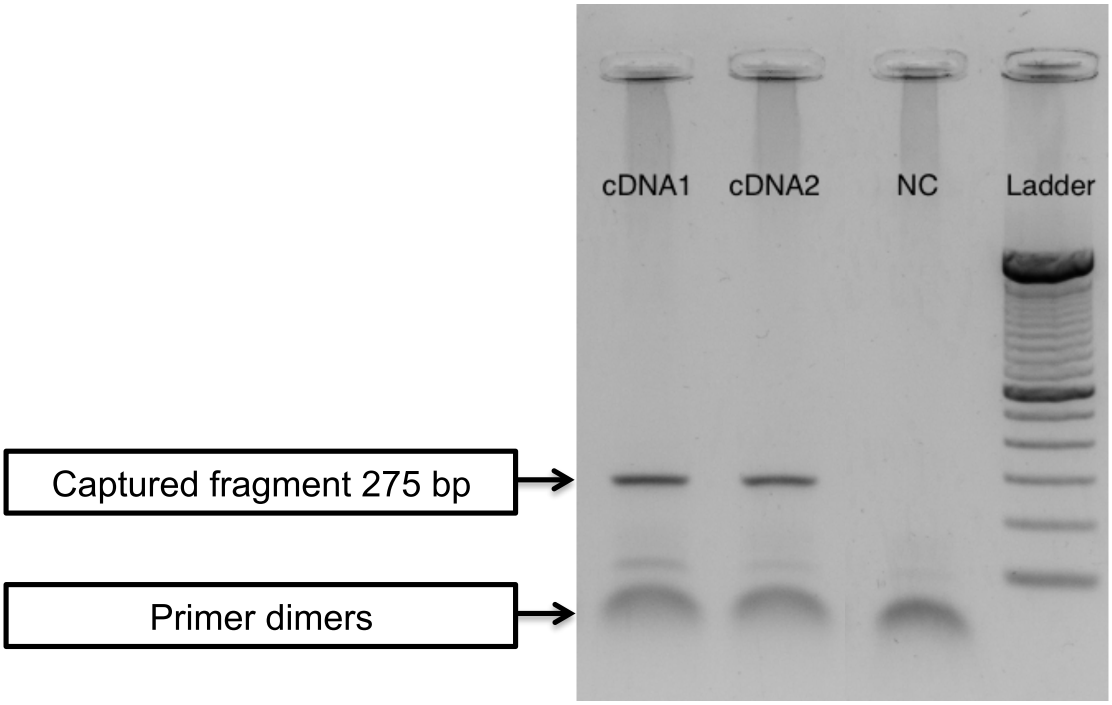

Supplementary Figure 16 Captured fragment of interest, size 275 bp (should be absent in the Negative Control NC) Primer dimers (will be removed by bead purification)

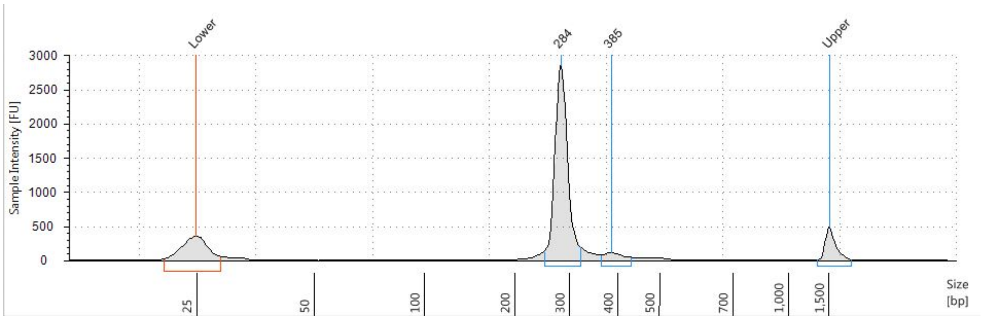

Supplementary Figure 17 Tapestation D1000 High sensitivity view of a finished smMIP library. Only the desired peak at 284 bp remained after bead purification.

Condition	cDNA amount (ng)	Replicate number	Number of PCR cycles	Pearson correlation	Pearson correlation p-value	Total number of UMIs	Total number of fragments matched to probe	Total number of fragments
**ERCC1**	10	1	20	0.96	7.4e-52	405505	887894	970618
**ERCC2**	10	1	20	0.96	2.6e-52	359043	1205685	1339589
**ERCC1**	10	2	20	0.96	4.4e-53	463487	1136389	1250081
**ERCC2**	10	2	20	0.96	1.8e-50	365281	1187664	1327331
**ERCC1**	50	1	20	0.95	8.1e-46	531747	1105532	1208478
**ERCC2**	50	1	20	0.95	2.3e-48	603747	1081440	1199556
**ERCC1**	50	2	20	0.94	1.2e-44	298229	957336	1054592
**ERCC2**	50	2	20	0.95	2.2e-47	563164	1093713	1204246

Supplementary Table 1 Statistics cDNA-smMIPs ERCC experiment. Pearson correlation is between prob e-level expression estimates and the known ERCC transcript concentrations.

Sample	Number of reads
CaptureSeq DE Sample 1/ERCC1	2,055,311
CaptureSeq DE Sample 2/ERCC1	4,172,480
CaptureSeq DE Sample 3/ERCC1	2,725,456
CaptureSeq DE Sample 4/ERCC1	3,188,557
CaptureSeq DE Sample 5/ERCC1	1,937,785
CaptureSeq DE Sample 1/ERCC2	1,470,603
CaptureSeq DE Sample 2/ERCC2	3,455,342
CaptureSeq DE Sample 3/ERCC2	2,558,009
CaptureSeq DE Sample 4/ERCC2	2,897,105

Supplementary Table 2 Number of reads for CaptureSeq ERCC experiment. Data was previously published[1].

Gene	Mean log2(1+FPKM)
**AIRE**	0.31
**ASCL1**	1.94
**GUSB**	5.52
**HPRT1**	5.69
**IFIT1**	4.33
**IFNG**	0.56
**IL23A**	1.90
**MYC**	5.92
**PTBP1**	6.18
**PTBP2**	2.44
**RUNX3**	5.81
**YWHAZ**	7.81

Supplementary Table 3 Mean expression (mean of log2(1+FPKM) values) in 660 RNA-Seq samples for EBV cell lines from the Geuvadis Project[2].

Experiment name	Individual id	cDNA amount (ng)	Replicate number	Experimentalist	Number of PCR cycles	Pearson correlation	Pearson correlation p-value	Total number of UMIs (corrected)	Total number of fragments matched to probe	Total number of fragments	Condition in Figure 2
**ebv-1**	EBV2	10	1	E1	21	0.95	2.89e-06	13565	141496	178877	EBV2 Exp.1 Rep.1
**ebv-1**	EBV2	10	2	E1	21	0.95	1.90e-06	12748	137484	175865	EBV2 Exp.1 Rep.1
**ebv-1**	EBV2	50	1	E1	21	0.95	1.42e-06	65771	742567	823000	EBV2 Exp.1 Rep.1
**ebv-1**	EBV2	50	2	E1	21	0.95	1.83e-06	64107	696125	775808	EBV2 Exp.1 Rep.1
**ebv-1**	EBV2	100	1	E1	21	0.95	2.39e-06	21442	251875	295220	
**ebv-1**	EBV2	100	2	E1	21	0.96	1.29e-06	60233	725666	800397	
**ebv-1**	EBV3	10	1	E1	21	0.94	5.41e-06	22943	233266	281287	EBV3 Exp.1 Rep.1
**ebv-1**	EBV3	10	2	E1	21	0.95	3.55e-06	23387	241113	288473	EBV3 Exp.1 Rep.1
**ebv-1**	EBV3	50	1	E1	21	0.95	2.39e-06	157386	1362619	1512590	EBV3 Exp.1 Rep.1
**ebv-1**	EBV3	50	2	E1	21	0.94	3.69e-06	124700	1098869	1214801	EBV3 Exp.1 Rep.1
**ebv-1**	EBV3	100	1	E1	21	0.95	2.81e-06	193478	1597529	1753395	
**ebv-1**	EBV3	100	2	E1	21	0.95	2.97e-06	234172	1404719	2472729	
**ebv-2**	EBV2	5	1	E1	21	0.90	7.33e-05	5385	300264	391367	
**ebv-2**	EBV2	5	1	E1	23	0.91	3.72e-05	4789	1026326	1332846	
**ebv-2**	EBV2	5	2	E1	21	0.94	8.13e-06	10265	333429	461444	
**ebv-2**	EBV2	5	2	E1	23	0.92	2.83e-05	5269	1076610	1381992	
**ebv-2**	EBV2	5	1	E2	21	0.92	2.11e-05	11297	417743	581397	
**ebv-2**	EBV2	5	1	E2	23	0.92	2.48e-05	11290	839598	1314672	
**ebv-2**	EBV2	5	2	E2	21	0.88	1.54e-04	11095	281171	393274	
**ebv-2**	EBV2	5	2	E2	23	0.92	2.22e-05	11844	785829	1155739	
**ebv-2**	EBV2	10	1	E1	21	0.91	3.27e-05	10631	357607	450660	EBV2 Exp.2 Rep.1
**ebv-2**	EBV2	10	2	E1	21	0.89	9.81e-05	14571	475533	570780	EBV2 Exp.2 Rep.1
**ebv-2**	EBV2	10	1	E2	21	0.91	4.09e-05	25161	407090	524927	EBV2 Exp.2 Rep.2
**ebv-2**	EBV2	10	2	E2	21	0.91	4.73e-05	23958	90174	114225	EBV2 Exp.2 Rep.2
**ebv-2**	EBV2	50	1	E1	21	0.89	9.94e-05	127040	1105632	1214179	EBV2 Exp.2 Rep.1
**ebv-2**	EBV2	50	2	E1	21	0.88	1.38e-04	177399	1630545	1783730	EBV2 Exp.2 Rep.1
**ebv-2**	EBV2	50	1	E2	21	0.88	1.59e-04	192929	1144032	1273042	EBV2 Exp.2 Rep.2
**ebv-2**	EBV2	50	2	E2	21	0.87	2.26e-04	190353	1130159	1328431	EBV2 Exp.2 Rep.2
**ebv-2**	EBV3	5	1	E1	21	0.89	1.00e-04	9991	579212	760060	
**ebv-2**	EBV3	5	1	E1	23	0.95	1.96e-06	8793	1526039	1900912	
**ebv-2**	EBV3	5	2	E1	21	0.92	2.41e-05	9829	659320	823459	

**ebv-2**	EBV3	5	2	E1	23	0.85	4.19e-04	10368	1748686	2140101	
**ebv-2**	EBV3	5	1	E2	21	0.81	1.28e-03	340	341	448	
**ebv-2**	EBV3	5	1	E2	23	0.95	2.34e-06	22190	1137359	1653467	
**ebv-2**	EBV3	5	2	E2	21	0.94	6.17e-06	23860	788347	1066562	
**ebv-2**	EBV3	5	2	E2	23	0.94	8.01e-06	23263	1187868	1661428	
**ebv-2**	EBV3	10	1	E1	21	0.95	1.78e-06	20049	799077	962161	EBV3 Exp.2 Rep.1
**ebv-2**	EBV3	10	2	E1	21	0.95	2.85e-06	31431	914849	1084015	EBV3 Exp.2 Rep.1
**ebv-2**	EBV3	10	1	E2	21	0.95	2.92e-06	42434	600107	773132	EBV3 Exp.2 Rep.2
**ebv-2**	EBV3	10	2	E2	21	0.95	2.80e-06	44222	655425	848198	EBV3 Exp.2 Rep.2
**ebv-2**	EBV3	50	1	E1	21	0.93	1.51e-05	216188	1493710	2776415	EBV3 Exp.2 Rep.1
**ebv-2**	EBV3	50	2	E1	21	0.93	9.08e-06	219824	1476727	2939897	EBV3 Exp.2 Rep.1
**ebv-2**	EBV3	50	1	E2	21	0.93	1.40e-05	238950	1451901	1754404	EBV3 Exp.2 Rep.2
**ebv-2**	EBV3	50	2	E2	21	0.94	7.39e-06	186026	1173710	1344338	EBV3 Exp.2 Rep.2

Supplementary Table 4 Statistics cDNA-smMIPs for the EBV experiments (continued from the previous page). Note that only the samples with 10 ng and 50 ng input were used for the analysis presented in the main text. The rightmost column contains the condition label as used in Figure 2a (Rep.1 and Rep.2 in the right column correspond with respectively experimentalist E1 and E2 in the fifth column). Pearson correlation is between the cDNA-smMIP gene-level expression estimates and log2(1+RPKM) expression estimates from the Geuvadis project[2].

Condition	cDNA amount (ng)	Replicate number	Pearson correlation	Pearson correlation p-value	Total number of UMIs (corrected)	Total number of fragments matched to probe	Total number of fragments
**Candida**	10	1	0.94	6.79e-06	4281	72161	111508
**Candida**	10	2	0.96	6.24e-07	4040	72819	115360
**Candida**	50	1	0.94	6.97e-06	18227	308001	372610
**Candida**	50	2	0.94	4.87e-06	18082	295139	358076
**Candida**	100	1	0.91	4.45e-05	3755	71194	109588
**Candida**	100	2	0.92	2.29e-05	3020	55906	91981
**RPMI**	10	1	0.72	8.26e-03	38	12862	49286
**RPMI**	10	2	0.92	2.38e-05	1972	37217	73138
**RPMI**	50	1	0.94	5.73e-06	3810	65312	104411
**RPMI**	50	2	0.91	5.06e-05	3454	57724	96380
**RPMI**	100	1	0.80	1.59e-03	331	11614	39831
**RPMI**	100	2	0.77	3.67e-03	128	6790	37378

Supplementary Table 5 Statistics cDNA-smMIPs for the PBMC stimulation experiments. Only the samples with 50 ng cDNA input were used for the analysis presented in the main text (Fig. 4). Pearson correlation is between cDNA-smMIP gene expression estimates and the previously published[3] RNA-Seq log2(1+RPKM) expression values.

Dilution step	Replicate number	Number of molecules (corrected)	Total number of fragments matched to probe	Total number of fragments
**1**	1	11346	1799628	2266296
**1**	2	13850	2193327	2705790
**2**	1	12190	1839732	2290528
**2**	2	13610	1994684	2508695
**3**	1	13519	1888932	2332757
**3**	2	13242	1901535	2356145
**4**	1	14020	1921078	2369718
**4**	2	15026	2097665	2570465
**5**	1	15931	2110537	2551972
**5**	2	14153	1970863	2424109
**6**	1	15162	2181406	2656728
**6**	2	13302	2032701	2488302
**7**	1	12820	1807516	2226198
**7**	2	13194	2077815	2545695
**8**	1	13939	1883085	2315611
**8**	2	12425	1993788	2425426
**9**	1	14778	2242090	2760953
**9**	2	15379	2013562	2507404
**10**	1	13154	2006712	2478182
**10**	2	14167	1940140	2390244

Supplementary Table 6 Statistics cDNA-smMIPs for allelic ratio experiment. A serial dilution of K562 cDNA with HEK293 cDNA was performed. The first and last dilution steps correspond to respectively only K562 cDNA and only HEK293 cDNA.

Amortizing costs	Total costs	Per sample	Per sample per gene
20 genes (100 smMIPs)	$790.00	$7.90 (100 samples)	$0.40
20 genes (100 smMIPs)	$790.00	$0.79 (1000 samples)	$0.04
**Fixed costs 20 genes (100 smMIPs)**	**Total costs**	**Per sample**	**Per sample per gene**
Reagents and plasticware (100 samples)	$321.53	$3.22	$0.16
Reagents and plasticware (1000 samples)	$3215.33	$3.22	$0.16
Sequencing reagents*§ (100 samples)	$649.75	$6.50	$0.32
Sequencing reagents*§ (1000 samples)	$6497.50	$6.50	$0.32
Capture and sequencing 100 samples	$971.28	$9.71	$0.48
Capture and sequencing 1000 samples	$9712.83	$9.71	$0.48
**Total cost summary 20 genes (100 smMIPs)**	**Total costs**	**Per sample**	**Per sample per gene (5 probes per gene)**
100 samples	$1761.28	$17.62	$0.88
1000 samples	$10502.83	$10.50	$0.53

Supplementary Table 7 Cost estimates for smMIP capture and sequencing (based on[4]).

*Assumes samples are sequenced on the Nextseq500 (Illumina) with 2x79bp runs

§ Assumes estimated average coverage is 10,000 reads/MIP/sample.

Component	Volume (µl)	Comment
**MIPs (0.5µl/ smMIP)**	47.5	For 95 cDNA-smMIPs
**T4 PNK**	1.9	*1 µl of T4 PNK enzyme per 25 µl of 100 µM MIP*
Subtotal	*49.4*	*Sum*
**H_2O**	4.6	
**10X T4 DNA ligase buffer with 10 mM ATP**	6	*10% of total volume*
**Total**	60	

Supplementary Table 8 Reaction mixture calculation for the phosphorylation of smMIPs used for the cDNA smMIP protocol.

Temperature	Time
**37 ˚C**	45 min
**65 ˚C**	20 min
**4 ˚C**	forever

Supplementary Table 9 Thermocycler protocol used for the smMIP phosphorylation.

Component	Volume for 30 reactions (µl)	Comment
**Ampligase DNA Ligase Buffer 10x**	75	
**MIP pool dilution**	9.9	smMIP pool 0.833µM diluted 1:625
**dNTP 0.25 mM**	0.96	
**Hemo Klentaq 10 U/µl**	9.6	
**Ampligase DNA Ligase 100 U/µl**	0.30	Critical: do not use less than 0.3 µl
**H_2O**	354.3	Add to make total volume
**Total mix**	450	15 µl/reaction
**cDNA (10 ng)**	10	
**Total**	25	µl/reaction

Supplementary Table 10 Reagent mixture example for smMIP capture of 30 samples.

Temperature	Time
95˚C	10 min
60˚C	forever*

Supplementary Table 11 Thermocycler protocol used for the smMIP capture, the capture is stopped after 18-24 hours.

Component	Volume for 1 reaction (μl)
EXO I (20.000 U/ml)	0.5
EXO III (100.000 U/ml)	0.5
Ampligase DNA Ligase Buffer	0.2
H_2O	0.8
Total	2

Supplementary Table 12 Reagent mixture for the smMIP exonuclease treatment.

Temperature	Time
37˚C	45 min
95˚C	2 min
4˚C	forever

Supplementary Table 13 Thermocycler protocol used for the smMIP exonuclease-treatment.

Component	Volume for 1 reaction (μl)
2X iProof	12.5
Illumina_PE_MIPBC_FOR* (100 μM)	0.125
Illumina_PE_MIPBC_REV BC1* (100 μM)	0.125
SYBR green* (100X in DMSO)	0.125
H_2O	7.125
Total PCR mix	20
Exonuclease-treated MIP sample	5
Total	25

Supplementary Table 14 Reaction mixture used for the Real-Time PCR. *Illumina_PE_MIPBC primer sequences are given in Supplementary Data 4.

Temperature	Time	
**98˚C**	30 sec	
**98˚C**	10 sec	
**60˚C**	30 sec	35 cycles
**72˚C**	30 sec	
**72˚C**	2 minutes	
**25˚C**	forever	

Supplementary Table 15: Qiagen Rotorgene RT-PCR protocol

**2X iProof**	**12.5**
**Illumina_PE_MIPBC_FOR* (100 μM)**	0.125
**H_2O**	1.125
**Total PCR mix**	13.75
**Illumina_PE_MIPBC_REV BC* (10 μM)**	1.25
**Exonuclease-treated MIP sample**	10
**Total**	25

Supplementary Table 16 Reaction mixture used for the PCR.

*Illumina_PE_MIPBC primer sequences are given in Supplementary Data 4.

Temperature	Time	
**98˚C**	30 sec	
**98˚C**	10 sec	# cycles determined by RT-PCR (21)
**60˚C**	30 sec	
**72˚C**	30 sec	
**72˚C**	2 minutes	
**4˚C**	forever	

Supplementary Table 17: Thermocycler protocol for the PCR

Custom Primer name	Primer sequence
**MIPBC_SEQ_FOR**	CATACGAGATCCGTAATCGGGAAGCTGAAG
**MIPBC_SEQ_REV**	ACACGCACGATCCGACGGTAGTGT
**MIPBC_SEQ_INDX**	ACACTACCGTCGGATCGTGCGTGT

Supplementary Table 18 Custom primers used for cDNA-smMIP sequencing

Name	Catalogue number	Supplier
**RNeasy Plus Mini Kit (250)**	74136	Qiagen
**iScript™ cDNA Synthesis Kit**	1708891BUN	Biorad
**QIAquick PCR Purification Kit (250)**	28106	Qiagen
**Elution Buffer EB**	1014608	Qiagen
**T4 PNK (10.000U/ml)**	M0201L	NEB
**10x T4 DNA ligase buffer**	B0202S	NEB
**10X Ampligase DNA Ligase Buffer**	A1905B	Epicentre/Illumina
**dNTPs (100mM set)**	10297018	Invitrogen/Thermo Fisher Scientific
**Hemo KlenTaq (10U/μl)**	M0332L	NEB
**Ampligase DNA Ligase (100U/μl)**	A0110K	Epicentre/Illumina
**EXO I (20.000U/ml)**	M0293L	NEB
**EXO III (100.000U/ml)**	M0206L	NEB
**2X iProof HF Master MIx**	1725310	Bio-Rad
**SYBR green (10.000x In DMSO)**	S-7563	Invitrogen/Thermo Fisher Scientific
**AmpureXP Beads**	A63881	Beckman Coulter
**Qubit® dsDNA HS assay kit**	Q32851	Invitrogen/Thermo Fisher Scientific
**Qubit® ssDNA assay kit**	Q10212	Invitrogen/Thermo Fisher Scientific
**Qubit® RNA assay kit**	Q32855	Invitrogen/Thermo Fisher Scientific
**Qubit® Assay Tubes**	Q32856	Invitrogen/Thermo Fisher Scientific
**QIAquick PCR Purification Kit (250)**	28106	Qiagen
**Tapestation D1000 Screentapes**	5067-5582	Agilent Technologies
**Tapestation D1000 Reagent**	5067-5583	Agilent Technologies
**Tapestation D1000 Ladder**	5067-5586	Agilent Technologies
**Rotorgene strip tubes and caps**	981106	Qiagen

Supplementary Table 19 Reagents used

Name	Catalogue number	Supplier
**DNA engine PCR machine**	PTC-0200	BioRad
**Rotor Gene Q**	9001550	Qiagen
**2200 Tapestation**	N/A	Agilent
**Qubit Fluorometer 2**	Q32866	Thermo Fisher Scientific

Supplementary Table 20 Equipment used.

Supplementary Methods

Bayesian hierarchical model to estimate differential expression

We constructed a statistical model to integrate observations from replicates into a single estimate of expression, and to quantify uncertainty in estimates of differential expression. We used a negative binomial distribution to model the unique molecule counts. We defined expression for a given probe as the logarithm of the mean of the negative binomial distribution; this expression is corrected for probe bias and normalized for sequencing depth. We assume that the overdispersion factor (relation between mean and variance) is the same for all probes. We allow for heterogeneity in the dispersion factor between experiments, as our results indicate that some experiments show more variability than others. The probe bias is estimated from differences in normalized counts (molecules per million molecules) between replicates and then used as a covariate in the model. We used Stan[5] to perform inference in this model using Markov chain Monte Carlo sampling. Stan was run independently for each condition (a condition is defined as a set of replicate experiments) to generate 1000 independent samples. We used these samples from the posterior distribution to estimate differential expression between conditions.

Mathematical formulation

To estimate differential expression for two conditions, we correct each pair of samples from the Markov chain for the probe bias using ordinary least squares regression.

We define M_{cr}^{p} as the number of molecules counted for probe p in condition c and replicate r. Thus, the pair of subscripts (c, r) thus uniquely defines an experiment, where $r = 1, \dots, R_c$, with R_c the number of replicates for condition c, and $c = 1, \dots, C$, with C the number of conditions, and $p = 1, \dots, P$, with P the number of smMIP probes. We define e_c^p as the expression value for probe p in condition c, corrected for probe bias $\mathbf{B}_p$ and normalized for differences in sequencing depth through library size factors S_{cr}. The counts for different replicates in the same condition

directly depend on this shared expression value. This is the mechanism by which observations from multiple replicates are combined to obtain accurate estimates.

The joint probability of the variables in the model conditional on fixed parameters is given by:

$$P(\mathbf{M}, \mu, \nu, \beta \mid \mathbf{B}, \mathbf{L}) = \prod_{c}\prod_{p} P\left(\mu_c^p\right) \prod_{r} P\left(M_{cr}^p \mid e_c^p, \nu_{cr}, \beta, B_p, L_{cr}\right) P\left(\beta_{cr}^j\right) P(\nu_{cr}) \tag{1}$$

The probe biases form a matrix B_j^p where j indexes the jth bias vector $(B_j^{p=1}, \dots, B_j^{p=P})$ estimated from the data as described below. β_{cr}^j is coefficient for the jth bias-vector on experiment (c, r) and is a random variable in the model, where we arbitrarily choose $\beta_{c,r=1}^j = 0$ for all j, c. We use the negative binomial distribution to model the smMIP molecule counts:

$$P(M_{cr}^p \mid e_c^p, \nu_{cr}, \beta, B_p, L_{cr}) = \mathrm{NB}(\mu_{rc}^p(e_c^p, \beta, B_p, L_{cr}), \left(\sigma_{cr}^p(\nu_{cr})\right)^2).$$

The expected value μ_{cr}^p for the negative binomial depends on the normalized expression value e_c^p, the library size factors and bias influences β as follows:

$$\mu_{cr}^p = \exp\left(e_c^p + L_{cr} + \sum_j \beta_{cr}^j B_j^p\right).$$

Thus, the influence (regression coefficient) of the probe bias B_j^p is modelled by a random continuous variable in the model. While we can infer influence of this bias from *differences* between replicate experiments, there is no information to decide which experiment actually was closest to the true expression value. Therefore we arbitrarily set the influence of the probe bias to zero for the first replicate in a given condition. However, this introduces an arbitrary offset to the normalized expression values e_c^p. As a consequence, when we estimate differential expression between two conditions, we again correct for the probe bias.

The mean-variance relationship for the negative binomial is given by

$$\left(\sigma_{cr}^{p}(\nu_{cr})\right)^{2} = \mu_{cr}^{p} + \frac{(\mu_{cr}^{p})^{2}}{\nu_{cr}},$$

where ν_{cr} is a hyperparameter with a Γ prior distribution:

$$P(\nu_{cr}) = \Gamma(\alpha = 1, \beta = \frac{1}{10})$$

As a result, the overdispersion relation is the same for all probes but may vary between experiments. This is in part necessary because of the shared mean between replicate experiments, which may increase variance in one replicate, and in part due to actual experimental variability between. Here our model differs from that of DESeq[6], which allows the overdispersion factor to vary between genes.

The library size factors L_{cr} are estimated from the molecule counts following the DESeq approach[6–8]:

$$\exp(L_{cr}) = \mathrm{median}_{p} \frac{M_{cr}^{p}}{\left(\prod_{c'r'} M_{c'r'}^{p}\right)^{1/N_e}},$$

where N_e represents the total number of experiments $N_e = \sum_c R_c$. As expression values e_c^p are to be compared between conditions they must be on a common scale. Therefore the size factors are estimated from all experiments jointly.

As we perform MCMC inference we do not optimize the nuisance parameters β and ν but integrate over the possible values consistent with the observations M and fixed parameters **B** and **S.**

Estimation of differential expression

The joint distribution fully factorizes into conditions c, as only constant parameters are shared between conditions. We therefore perform MCMC inference independently for each condition. This generates samples $s = 1, \ldots, S$ with S the number of MCMC samples (we used 1000 independent samples). A naive estimate of

differential expression between conditions $c = 1$ and $c = 2$ would consist of taking the average difference across samples:

$$\Delta^{p}_{\text{uncorrected}} = \frac{1}{S}\sum_{s}(e^{p,s}_{c=1} - e^{p,s}_{c=2}).$$

However, because we have set an arbitrary offset to the expression levels by setting the probe bias influence to zero for the replicate, $\beta_{c,r=0} = 0$, we need to again correct differential expression estimates for the possible presence of probe bias. For computational efficiency we obtain an estimate of differential expression $\Delta^{p,s}$ for each MCMC sample s by correcting for the probe bias vectors $\mathbf{B}_j$ using ordinary least squares regression (without overall mean effect) with different probes p as observations:

$$e^{p,s}_{c=1} = e^{p,s}_{c=2} + \Delta^{p,s}_{\text{corrected}} + \sum_{j} \beta'^{s}_{j} B^{p}_{j} + \varepsilon^{p}_{s},$$

where $\varepsilon^{p}_{s} \sim N(0, \sigma_s)$ is the unexplained error, and β'^{s}_{j} are the regression coefficients for each bias vector j and sample s. Thus, the β'_j can be estimated from the $p = 1, \ldots, P$ probe-level expression estimates. The differential expression estimate for probe p is then given by the corrected differential expression averaged over samples:

$$\Delta^{p} = \frac{1}{S}\sum_{s} \Delta^{p,s}_{\text{corrected}}$$

We used the samples $\Delta^{p,s}_{\text{corrected}}$ from the posterior distribution $P(\Delta^{p} \mid M)$ to quantify uncertainty in the estimates of differential expression.

Although technically it would be preferable to jointly model all conditions in a single model, we found that in practice this resulted in slow convergence and poor performance.

Estimation of probe bias

We used an empirical Bayes approach to estimate the values for the probe bias matrix B_j^p. The idea is to compare the difference in log-normalized counts between one pair of replicates with the difference in log-normalized counts between another independent pair of replicates. When the correlation across probes of these differences is very high, we assume we have identified a systemic probe bias.

First, we normalize the molecule counts by the total number of molecules counted for an experiment (c, r): $m_{cr}^p = \log\left(\frac{M_{cr}^p}{\sum_p M_{cr}^p}\right)$. Second, we compute the difference v_{c,r_1,r_2}^p between all pairs of replicates r_1, r_2 belonging to the same condition c:

$$v_{c,r_1,r_2}^p = m_{c,r_1}^p - m_{c,r_2}^p, \forall\ c \text{ and } \forall r_1, r_2 \in c, r_1 \neq r_2$$

Thus, we never compare replicates from different conditions. The number of vectors $\mathbf{v}_{c,r_1,r_2}$ is then given by $\sum_c\ R_c\,(R_c - 1)/2$ (with R_c the number of replicates for condition c). We then calculate the Spearman rank correlation between all pairs of vectors $\mathbf{v}_{c,r_1,r_2}$ for non-overlapping pairs of replicates (c, r_1, r_2) and (c', r'_1, r'_2) (i.e., $c, r_1 \neq c', r'_1$ and $c, r_1 \neq c', r'_2$ and vice versa). The underlying assumption is that differences between replicate experiments that are also present in independent experiments reflect systematic experimental artefacts.

Finally, we use the average of pairs of vectors $\mathbf{v}$ that have the highest correlations to define the matrix B_j^p, under the constraint that the set of replicates to define respectively the first vector and second vector are non-overlapping. For example, if a set of experiments consists of two conditions $c = 1,2$ and two replicates per condition, there are only two pairs of replicates that can be compared and consequently one probe bias vector B_j^p. We then choose the bias vector as follows:

$$B_{j=1}^p = \frac{1}{2}\left(v_{c=1,r_1=1,r_2=2}^p + v_{c=2,r_1=1,r_2=2}^p\right)$$

cDNA-smMIP experimental procedures

The cDNA smMIP experimental procedure described below is largely based on the smMIP protocol developed for genomic DNA[4,9]. All reagents used are specified in Supplementary Table 19; all equipment used is specified in Supplementary Table 20.

1) MIP pooling

We used three independent cDNA-smMIP pools for experiments testing differential expression of ERCCs (337 smMIPs), differential expression of EBVs and PBMCs (95 smMIPs), and allele specific expression (64 smMIPs). The detailed protocol below describes the experiment we used to measure differential expression with EBVs. In these experiments 5 µl of each of the 95 smMIPs were pooled into one single tube.

2) MIP Phosphorylation

An aliquot representing 0.5 µl per smMIP of the 1x pool was used for phosphorylation. Volumes of other components were calculated according the example in the Supplementary Table 8.

If necessary split the volume into more PCR tubes, not exceeding 100 µl/tube for optimal thermal conditions; Run the PCR program shown in Supplementary Table 9 on a PCR machine:

Calculate the concentration of the MIP pool:

$Vi \times Ci = Vf \times Cf$

In this example:

$0.5\ \mu l \times 100\ \mu M = 60\ \mu l \times Cf$

Vi= initial volume per MIP used for phosphorylation (=0.5 in example of Supplementary Table 8)

Ci= initial concentration per MIP (=standard of 100 µM)

Vf= volume of MIP pool (=60 µl in example of Supplementary Table 8)

Cf =concentration of the MIP pool in µM

(initial concentration x initial volume) / (Final volume) = final concentration

100 µM*0.5 µl/60 µl = 0.833 µM

Stopping point: If not proceeding to the next step, store phosphorylated MIP pool at 4ºC. Phosphorylated MIP pool can be used for multiple MIP captures. For production scale aliquot 1:10 dilutions and store at -20ºC to avoid multiple freeze-thaw cycles.

3) MIP Capture

The previously published protocol for genomic DNA[4] describes a MIP to gDNA molecule ratio of 800:1, corresponding to 264,000 MIP molecules per ng gDNA. For cDNA smMIPs we used 10-fold more smMIPs, to compensate for the higher amount of RNA than DNA molecules per cell, i.e. 2,640,000 smMIPs per ng cDNA.

1. Pipette 10 ng of cDNA in 10 µl (H_2O) in plate or strip tube; add a blanc with 10 µl H_2O. The cDNA input amount may differ per celltype, for several samples we used 3 different input amounts, e.g. 1 ng, 10 ng, 50 ng.
2. Make a MIP capture mix for at least 30 reactions (example in Supplementary Table 10).
3. Add 15 µl of the mix to 10 ng cDNA in 10 µl.
4. Make sure to change the lid off-set temperature in the thermocycler to 10˚C and run the PCR program shown in Supplementary Table 11.
5. Capture the MIPs for 18-24 hours at 60˚C, after that take samples from PCR machine and cool on ice to stop the reaction. Immediately continue with step 4 'Exonuclease-treatment'.

4) Exonuclease treatment

1. Cool the samples on ice after capture.
2. Make an exonuclease-treatment mastermix (Supplementary Table 12) for the amount of samples you have.
3. Add 2 µl of the mastermix to each captured sample.
4. Run the program in Supplementary Table 13 on a PCR machine.

Stopping point: If not proceeding to the next step, store exonuclease-treated MIP capture at 4ºC, for long-term store at -20ºC. Exonuclease-treated MIPs are sufficient for several PCR's.

5) Real Time PCR (using Qiagen Rotorgene)

Note: Real-Time PCR is used to determine the amount of PCR cycles needed for sufficient amplification; RT-PCR can be used for sequencing if machine can be paused after appropriate amplification; or PCR is repeated with determined amount of cycles.

Make a PCR mastermix according to Supplementary Table 14.

1. Fill out 20 µl to each reaction tube.
2. Add 5 µl exonuclease-treated sample or negative control (captured H_2O)
3. Vortex and spin down.
4. Transfer mixture in Qiagen strips for Rotorgene and carefully close with corresponding caps.
5. Run the PCR program shown in Supplementary Table 15 on Qiagen Rotorgene RT-PCR machine. Supplementary Figure 15 shows an example of a result.

6) PCR

1. Make a PCR mastermix according to Supplementary Table 16. Always prepare 10% extra.
2. Fill out 13.75 µl PCR mastermix in PCR strip tube or plate.
3. Add 1.25 µl reverse BC primer to barcode each sample.
4. Add 10 µl exonucelase-treated MIP sample.
5. Run the PCR program shown in Supplementary Table 17.
6. Check PCR product (2 µl) on agarose gel (Supplementary Figure 16).

Stopping point: If not proceeding to the next step store PCR products at 4ºC, for long-term store at -20ºC.

7) Purification with AmpureXP Beads

Before you start: Shake bead bottle thoroughly since beads settle overnight. Aliquot your volume in an eppendorf tube and let that adjust to room temperature around ~30 minutes. Prepare freshly made 70% ethanol.

1. Pool equal amount of each PCR sample. Don't add the blanc to the pool. (maximum of 1 PCR plate = 96 samples per Eppendorf tube)

   Note: Standard pooling of a plate is 5 µl per sample when similar intensities are seen on gel for all samples, when using less samples for instance when running the MIP assay for the first time on 4 controls, pool 10-15 µl each.

   OPTIONAL: pool more or less PCR product according to intensity of the agarose gel bands

2. Add Ampure XP beads (decide volume from gel image or tapestation 0.85x)
3. Vortex, spin down and incubate 10 minutes on room temperature.
4. Incubate tube 5 minutes on the magnetic rack.

5. Transfer volume to new tube (beads contain DNA, store tube until you verified the results)
6. Wash with 700 µl 70% ethanol, slightly invert the tube for 30 seconds.
7. Discard the ethanol, without touching the beads.
8. Repeat step 6 and 7
9. Leave tube open on magnetic rack to dry the beads.
10. Add 25-50 µl low TE (depending on the amount of samples pooled 4-96) for elution, vortex and spin tube.
11. Incubate at least 1 minute on magnetic rack.
12. Transfer library in new tube.
13. Verify your results on Tapestation D1000 High Sensitivity. If not all extra peaks are gone, repeat purification (Supplementary Figure 17)
14. Determine final concentration by Qubit (HS) measurement.

Stopping point: Store purified pool at 4ºC until ready for sequencing. For long-term store at -20ºC.

20 µl of a 4 nM library was used for sequencing on the Illumina Nextseq500.

8) Nextseq500 sequencing of prepared cDNA smMIP library

Sequencing of smMIP libraries requires primer require spike-in of custom primers as described previously[4]. Add 9 µl of custom primer "MIPBC_SEQ_FOR" to cartridge position 20 (Read1); 9 µl of custom primer "MIPBC_SEQ_REV" to cartridge position 21 (Read2), and 9 µl of custom primer "MIPBC_SEQ_INDX" to cartridge position 22 (Index Read1) each with a clean pipette tip.

The run is performed with 2x80 cycles, i.e. 2x79bp paired-end reads, and an 8bp index read. Custom primer sequences as published previously were used (Supplementary Table 18; IDT, 100µM, IDTE buffer).

Supplementary References

1. Clark, M. B. *et al.* Quantitative gene profiling of long noncoding RNAs with targeted RNA sequencing. *Nat. Methods* **12,** (2015).
2. Lappalainen, T. *et al.* Transcriptome and genome sequencing uncovers functional variation in humans. *Nature* **501,** 506–5011 (2013).
3. Smeekens, S. P. *et al.* Functional genomics identifies type I interferon pathway as central for host defense against Candida albicans. *Nat Commun* **4,** 1342 (2013).
4. O'Roak, B. J. *et al.* Multiplex targeted sequencing identifies recurrently mutated genes in autism spectrum disorders. *Science* **338,** 1619–22 (2012).
5. Carpenter, B. *et al.* Journal of Statistical Software Stan : A Probabilistic Programming Language. *J. Stat. Softw.* **VV,** (2016).
6. Anders, S. & Huber, W. Differential expression analysis for sequence count data. *Genome Biol.* **11,** R106 (2010).
7. Oshlack, A., Robinson, M. D. & Young, M. D. From RNA-seq reads to differential expression results. *Genome Biol.* **11,** 220 (2010).
8. Robinson, M. D. & Oshlack, A. A scaling normalization method for differential expression analysis of RNA-seq data. 1–9 (2010). doi:10.1186/gb-2010-11-3-r25
9. Hiatt, J. B., Pritchard, C. C., Salipante, S. J., O'Roak, B. J. & Shendure, J. Single molecule molecular inversion probes for targeted, high-accuracy detection of low-frequency variation. *Genome Res.* **23,** 843–54 (2013).
